# Prevalence of chronic venous insufficiency and deep vein thrombosis in cirrhotic patients

**DOI:** 10.3389/fmed.2023.1214517

**Published:** 2023-09-27

**Authors:** Leonardo da Cruz Renó, Francisco Tustumi, Daniel Reis Waisberg, Vinicious Rocha Santos, Rafael Soares Pinheiro, Rubens Arantes Macedo, Lucas Souto Nacif, Liliana Ducatti, Rodrigo Bronze De Martino, Alexandre Maximiniano Trevisan, Luiz Carneiro D’Albuquerque, Wellington Andraus

**Affiliations:** Department of Gastroenterology, School of Medicine, University of São Paulo, São Paulo, Brazil

**Keywords:** venous incompetence, liver disease, deep vein thromboembolism, cirrhosis, venous disease

## Abstract

**Summary:**

People with cirrhosis of the liver are at risk for complications that can worsen their quality of life and increase morbidity and mortality. Contrary to previous beliefs, cirrhosis does not protect against the development of thromboembolic events, and cirrhotic patients may have higher rates of deep vein thrombosis (DVT).

**Background and aims:**

The study of chronic venous disease and its impact on patients with cirrhosis is unknown in the literature and may be an important fact since this condition also had impact on quality of life and morbidity. The aim of this study is to evaluate the prevalence of DVT (Deep Venous thrombosis) in outpatients with cirrhosis and the degree of chronic venous insufficiency, evaluating possible correlations between clinical and laboratory aspects of cirrhotic patients with these pathologies.

**Methods:**

Patients with cirrhosis were evaluated in the outpatient clinic of the Liver Transplantation and Hepatology Service of HC-FMUSP from November 2018 to November 2022, with clinical evaluation, venous disease questionnaires, data collection of imaging and laboratory tests, and venous color Doppler ultrasound. The information was analyzed by the University of São Paulo (USP) Statistics Department.

**Results:**

There was a prevalence of 7.6% of DVT in studied patients, VCSS score 6.73 and severe CEAP classification (C4-6) 32.1%. There was no association of DVT with qualitative variables by the Fisher test such as Child Turcotte Pugh Scale (CTP) (*p* = 0.890), dichotomized INR values (*p* = 0.804), etiology of cirrhosis (*p* = 0.650) and chronic kidney disease (*p* > 0.999), nor with quantitative variables by t-student’s such as age (*p* = 0.974), Body Mass Index (BMI) (*p* = 0.997), MELD score (*p* = 0.555), Albumin (*p* = 0.150) and Platelets (*p* = 0.403). We found that as the severity of ascites increases, there is an increase in the proportion of patients classified in the category indicating more severe clinical manifestations of chronic venous disease (C4 to C6). The mean age (54 years) was higher in patients with DVT than in those without. The mean BMI of patients without DVT (25.7 kg/m^2^) is lower than that of patients with DVT (27.0 kg/m^2^). The prevalence of DVT is higher in patients with thrombophilia (20.0%) than in those without (7.0%). This suggests an association between the two variables. The descriptive measures of the MELD score, the cirrhosis scale used for liver transplant waiting lists, did not indicate an association of this scale with the occurrence of DVT.

**Conclusion:**

The incidence of VTE (Venous Thromboembolic Events) and CVD (Chronic Venous Disease) within the sample surpassed that of the general population; nevertheless, more studies are required to validate these results. Concerning venous thromboembolism, no correlation was observed between the variables within the sample and the augmented risk of VTE. Regarding chronic venous disease, studies have shown that edema and orthostatism are correlated with increased severity of CVD on the VCSS scales. Statistical dispersion methods suggest that patients with higher BMI and more severe liver disease (according to the Child-Pugh score) are more likely to experience worsening of CVD. About chronic venous disease, studies have shown that edema and orthostatism are correlated with increased severity of CVD on the VCSS scales.

## Highlights

- Prevalence of 7.6% of DVT.
- Venous Clinical Severity Score (VCSS) score 6.73.
- C4-6–Clinical signs, Etiology, Anatomic distribution, Pathophysiologic condition (CEAP) classification 32.1%.

## Introduction

The liver plays a key role in coagulation, and liver disease, by causing profound changes in the synthesis of coagulation factors, altering the hemostatic balance with a reduction in both pro- and anticoagulant factors. This imbalance can lead to potentially fatal clinical changes due to hemorrhagic or thromboembolic states (1, 2). For decades, it was believed that the increase in International Normalized Ratio (INR) associated with thrombocytopenia in patients with advanced liver disease was a form of self-anticoagulation that protected these patients from thromboembolic episodes, but in recent years there have been changes in the understanding of coagulation mechanisms. The importance of the endothelium, platelets, and the reticuloendothelial system as mediators in the phases of thrombus formation, thrombus limitation and lysis (cell theory) has been observed and, on the contrary, studies show that there may be an increase in cases of thromboembolism in these patients (3). The incidence of venous thromboembolism in patients with cirrhosis ranged from 0.5 to 8.1% in different studies, according to a systematic review by Aggarwal et al., (1), 3.7% according systematic review of Ambrosino et al. (2) highlighting the importance of clinical suspicion in these patients and evaluating the benefits of prophylactic regimens. Qi et al. (4) performed a systematic review of 20 studies when they evaluated the incidence and prevalence of VTE using random effect models and analyzed subgroups by type of VTE [deep vein thrombosis (DVT), pulmonary embolism (PE)], type of liver disease (cirrhosis alone/unclassified liver disease or non-cirrhotic), region in which the study was conducted (USA/Europe/Asia), total number of patients observed with liver disease (greater or less than 1000), study quality (high/low), and method of case identification (ICD codes/clinical charts). The incidence of VTE ranged from 0.33 to 6.32% in 14 studies with a pooled value of 1% [95% confidence interval (CI) 0.7–1.3%]. The pooled incidence of DVT and PE was 0.6% (95% CI 0.4–0.8%) and 0.28% (95% CI 0.13–0.49%), respectively. The prevalence of VTE ranged from 0.6 to 4.69% in six studies, with a pooled prevalence of 1.0% (95% CI 0.7–1.2%). The pooled prevalence of DVT and PE was 0.7% (95% CI 0.6–0.9%) and 0.36% (95% CI 0.13–0.7%), respectively. Venous thromboembolism, with its potentially fatal consequences, is a major health problem worldwide, with at least 200,000 new cases per year in the United States and an incidence of 74.5 per 100,000 people per year in the United Kingdom. The global incidence of deep vein thrombosis is 5 cases per 100,000 people per year, and the risk of developing the disease varies with age, affecting approximately 2 to 3 cases per 100,000 people between the ages of 30 and 49 and approximately 20 cases per 100,000 people between the ages of 70 and 79 (5, 6).

The Virchow’s triad implicates three main factors thought to contribute to VTE: hypercoagulability, endothelial damage, and venous stasis. Liver diseases potentially thrombogenic due to venous stasis caused by ascites, sarcopenia with immobility and high intrabdominal pressure resulting in venous stasis, as well as invasive procedures leading to inflammation and infectious conditions, which are other factors of Virchow’s triad (7). Chronic venous insufficiency is defined as a dysfunction of the venous system caused by valvular incompetence associated or not with obstructed venous flow. It may affect the superficial venous system, the deep venous system, or both. In addition, venous dysfunction may be congenital or acquired (8).

Approximately 7 million people in the United States have chronic venous insufficiency. It is the cause of 70 to 90 percent of all leg ulcers (9).

### Coagulation mechanism in liver disease

In patients with cirrhosis, because there are changes in coagulation proteins, with an increase in Prothrombin Activity Time and Activated Partial Thromboplastin Time, it was believed that there was protection against DVT and Pulmonary Thromboembolism (PE), but studies show that these data are not real, possibly because other procoagulant proteins may be at normal levels or increased, factors that inhibit thrombus formation may be reduced (protein C, protein S), in addition to other prothrombotic factors present in these patients (10).

Apart from Von Willebrand factor, Tissue Plasminogen Activator (TPA), Plasminogen Activator Inhibitor-1 (PAI-1), Thrombomodulin (TM), and Plasminogen Activated Urokinase (Upa), the liver synthesizes most coagulation factors (11).

**FIGURE 1:**
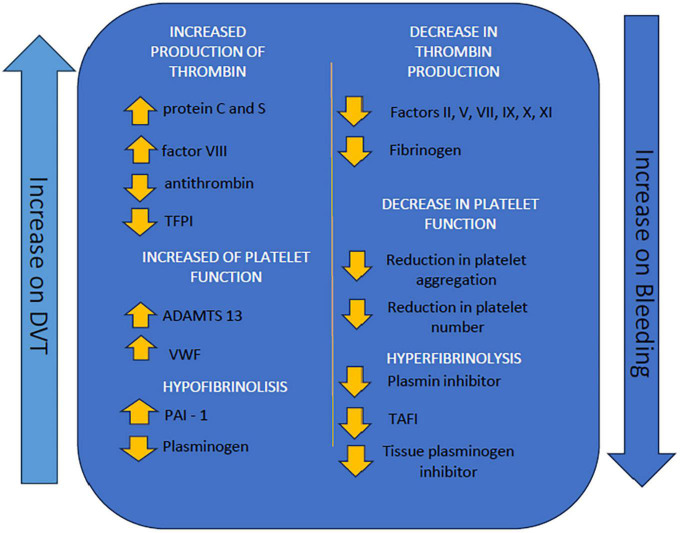
Illustrative scheme demonstrating factors that lead to greater coagulability or greater hemorrhagic tendency in patients with cirrhosis (14). TFPI, Tecidual factor pathway inhibitor; ADAMTS-13, a disintegrin and metalloprotease with thrombospondin type 1 repeats 13; VWF, Von Willembrand factor; PAI-1, plasminogen inhibitor 1; TAFI, Thrombin activatable fibrinolysis inhibitor.

Factor VIII is typically elevated in patients with cirrhosis, somewhat offsetting the decrease in other coagulation factors. At the same time, the procoagulants PtnC, PtnS, and antithrombin are decreased. However, normal thrombin generation activity has been demonstrated, as well as increased levels of Von Willebrand factor with greater platelet activation, although these are often reduced (12). The preservation of thrombin generation in cirrhosis is probably due to a rebalancing of coagulation mechanisms caused by the reduction of procoagulants (except factor VIII and Von Willebrand factor) and naturally occurring anticoagulants. The increase in Von Willebrand factor may also contribute to the increase in factor VIII by binding to it and preventing its cleavage and elimination (13). This results in acquired resistance to the anticoagulant action of thrombomodulin due to increased levels of factor VIII and decreased levels of its main physiological inhibitor, protein C (11, 12) Figure 1.

### Venous insufficiency

Venous insufficiency usually results from venous hypertension secondary to valvular reflux and/or venous obstruction. Despite the wide variety of signs or symptoms associated with chronic venous insufficiency, the presence of venous hypertension appears to be very common. Venous hypertension can be caused by primary valve reflux or secondary to previous cases of DVT, and other causes of venous hypertension obstruction occur in obesity due to high abdominal pressure or failure of the calf muscle pump as occurs after ankle trauma, fibrosis secondary to chronic local inflammation, and severe loss of muscle mass. Reflux may occur in the superficial, deep, or both venous systems (15). Endothelial inflammation is known to be a major contributor to the development of CVD, which is perpetuated by venous hypertension and valvular insufficiency. Changes in shear stress affect endothelial cells, leading to their activation and recruitment of leukocytes with the release of pro-inflammatory agents.

The damaged endothelium remains a trigger for the ongoing inflammatory cascade leading to venous and valve wall damage and worsening CVD. Endothelial dysfunction is a central aspect linking chronic venous disease and DVT, for which endothelial dysfunction is also a risk factor (16).

## Venous disease classifications

### CEAP classification

The CEAP classification today is universally adopted to provide an orderly framework on venous pathology for communication between colleagues and decision-making. The use of this classification allows an organized categorization of the key elements of venous disease in each case and the interrelationships between clinical manifestations, causes and anatomical distribution. It describes static components that do not change with the response to treatment and are not used with the severity score of chronic venous disease, for this the American Venous Forum recommends the use of other scales and today the Villalta and the VCSS are the most used (17–19).

In the CEAP classification, clinical signs are divided into 7 classes ranging from C0 to C6, with C0 being total absence of signs related to venous disease and C6 being active venous ulcer (Supplementary material).

### VCSS–Venous clinical severity score

The system of increasing classification of severity, proposed by *the American Venous Forum (Venous Clinical Severity Scale—VCSS)*, consists of ten clinical descriptors: pain, presence of varicose veins, venous edema, skin pigmentation, induration (fibrosis, hypodermitis, white atrophy, lipodermatosclerosis), inflammation (erythema, eczema dermatitis), presence, diameter and duration of active ulcer and use of compressive therapy (20) (Supplementary material).

### Primary endpoint

Measurement of the prevalence of deep vein thrombosis in patients with cirrhosis at the HCFMUSP gastroenterology outpatient clinic.

### Secondary objectives

Evaluation of the degree and presence of chronic venous disease in patients with cirrhosis from the outpatient gastroenterology service of the HC-FMUSP.

### Patients and methods

#### Ethical aspects

After receiving approval from the CEP of HC-FMUSP, a cross-sectional study aimed to assess the prevalence of venous disease in patients with cirrhosis. All patients provided their signature on the informed consent form. The HC-FMUSP CEP adopted this model to fulfill the requirements set forth by Resolution 196/96 of the National Health Council (CNS).

#### Population studied

Each patient was evaluated only once to verify the presence of venous disease. A total of 119 patients met all study inclusion criteria. We studied patients with cirrhosis diagnosed by the HC-FMUSP hepatology team using clinical, imaging, and laboratory criteria, all from the HC-FMUSP Liver Transplant, Ascites, and Hepatology Outpatient Clinic of the Gastrointestinal Surgery Service. Data collection started in November 2018 and ended in November 2022. In 2018, 03 patients were evaluated, in 2019, 40 patients, in 2020, 35 patients, in 2021, 27 patients, and in 2022, 27 patients were evaluated for a total of 119 patients. None of these patients developed DVT after evaluation. All patients with a history of DVT when already with clinically significant liver disease were DVT.

#### Inclusion criteria

Outpatients in the Liver Transplantation Outpatient Clinic and in the Ascites and Hepatology Clinics of the HCFMUSP (Hospital das Clínicas da Faculdade de Medicina da Universidade de São Paulo–Brazil) with a confirmed diagnosis of cirrhosis.

#### Exclusion criteria

Patients who did not agree to sign the Free and Informed Consent Form were excluded.

#### Development of the study

This is a single-center observational study in which data collection, clinical examination, and venous Doppler ultrasound were performed by the same investigator. The medical record was used to collect laboratory and imaging data. Clinical assessment and physical examination of the lower extremities were performed to verify the functional status of the venous circulation. Classification was performed according to internationally established criteria (21). The physical examination was first performed with the patient in the orthostatic position. It was designed to evaluate edema, the presence of lipodermatosclerosis or hyperpigmentation, varicose veins, and active or healed ulcers. Patients signed the free and informed consent form and assisted in completing the anthropometric, demographic, and clinical data evaluation form. The clinical evaluation was performed immediately before or after the physical examination and consisted of specific standardized questionnaires to assess the integrity of the lower extremity venous system, a hepatic insufficiency scale, activities of daily living scales, and questionnaires to assess pain, discomfort, and disability related to varicose veins (22).

Questionnaires included the Karnofsky Performance Scale; the Child-Turcotte-Pugh Scale (CTP); and the VCSS Scale.

Doppler ultrasound was initially performed in the dorsal decubitus position to evaluate the inferior vena cava and iliac veins, the common, superficial, deep, and popliteal femoral veins for patency, presence or absence of reflux, venous wall thickness, presence, or absence of intraluminal fibrosis or partially recanalized thrombosis, and the patient was then placed in the orthostatic position for distal compression maneuvers to evaluate venous reflux and the superficial venous system. The internal and external saphenous veins were scanned transversely and longitudinally for diameter measurement, assessment of pathologic reflux, and signs of previous or recent phlebitis. The popliteal, muscular, posterior, anterior tibial, and fibular veins were also evaluated in the orthostatism. Laboratory data for the dates closest to the time of data collection were obtained from medical records. BMI was calculated according to World Health Organization (WHO) criteria (23).

#### Statistical analysis

We performed a descriptive analysis of the data to characterize the patient profiles and summarize the results of the study. Then, appropriate statistical models were considered to estimate the prevalence and identify the risk factors for deep vein thrombosis and chronic venous insufficiency. The mean, standard deviation, median, minimum, maximum, and first and third quartiles were calculated. Fisher’s exact test was used to evaluate the association between DVT and the qualitative variables. The *p*-values indicate that there is no association between any of the risk factors and the occurrence of DVT.

The associations of DVT with the quantitative variables: age, BMI, Meld score, albumin, and platelets, were evaluated using the t-Student’s test (24), and the results obtained indicate that there is no difference in the means of the variables in the two categories of DVT.

The Kruskal-Wallis test was used to evaluate the association between the VCSS and each of the qualitative variables (25). A non-parametric approach was used because the descriptive analysis of the variables showed that the distributions of the VCSS in the categories of categorical risk factors may not be normal, with the same variance in all categories.

A logistic regression model was adjusted (26) to study the association between the occurrence of DVT and the risk factors together. Automatic backward and forward variable selection methods were used to fit the model (27). However, in both backward and forward selection, no variable was selected that contributed significantly to the explanation of the occurrence of DVT. A LASSO model was also fitted (25), but no variable was selected.

The associations between the VCSS scale and the quantitative variables of age, BMI, and Meld Score were assessed by calculating Spearman’s correlation coefficient (28). The coefficient values and corresponding *p*-values shown in Table 10. A total of 73 indicate that there is no correlation between the VCSS and the quantitative variables individually. The statistical software RStudio for Windows (version 2022.07.2 + 576) was used for analysis.

Multiple linear regression models were then adjusted (29), with VCSS as the response variable and risk factors as explanatory variables.

## Results

Baseline characteristics are summarized in Table 1. The mean body mass index is 27 kg/m^2^. The first quartile of the Karnofsky Performance is equal to 60. This means that less than 25.0% of the patients are unable to perform manual and/or domestic activities. A total of 61.2% of patients did not consume alcohol. A total of 63% of the patients did not smoke and 58.0% had no previous abdominal or hernia surgery.

**TABLE 1:** Baseline characteristics.

	*N*	Mean ± Dp	Median (Min–Max)
Age	119	53.00 ± 13.23	57 (21–76)
BMI	115	27.00 ± 5.07	25.7 (19.5–40.4)
MELD	119	14.77 ± 4.5	15 (6–25)
Albumin	119	3.30 ± 0.79	3.4 (0.7–5)
Urea	118	46.0 ± 35.0	35 (10–232)
Platelets	118	99.5 ± 64.0	89 (10–299)
INR	119	1.41 ± 0.43	1.25 (0.9–3.6)

DVT was present in 9 (7.6%) patients. A total of 5 (4.2%) patients had thrombophilia. Child-Pugh score was classified as category B in most patients (58%). The median Model for End-Stage Liver Disease (MELD) score was 15.

### Deep vein thrombosis (DVT)

In this study, we found alcoholic cirrhosis in 23.5%, NASH in 22.7%, autoimmune hepatitis in 16.8%, and viral hepatitis in 12.6%, with DVT found in 7.1% of patients with alcoholic cirrhosis, 7.4% of patients with NASH, 16.8% of patients with autoimmune hepatitis, and 7.7% of patients with viral hepatitis. We evaluated the association between DVT and possible risk factors such as thrombophilia, Child-Pugh, MELD score, cirrhosis etiology, chronic kidney disease, and International Normalized Ratio (INR). Fisher’s exact test was used to evaluate the association between DVT and the qualitative variables. The results obtained are presented in Table 2. The *p*-values indicate that there is no association between any of the risk factors and the occurrence of DVT.

**TABLE 2:** Results of fisher’s test applied to evaluate the occurrence of association between DVT and risk factors (qualitative variables).

Variable	*p*-value
Thrombophilia	0.330
Child-Pugh	0.890
Etiology	0.650
CKD	>0.999
INR	0.804

The relationships between DVT and the quantitative variables presented in Table 3, indicate that there is no difference in the means of the variables in the two categories of DVT and in the association with the risk facts in the population of patients with cirrhosis.

**TABLE 3:** Results of student’s *t*-test applied to evaluate association between DVT with risk factors in patients with cirrhosis (quantitative variables).

Variable	*p*-value
Age	0.974
BMI	0.997
MELD score	0.555
Albumin	0.105
Platelets	0.403

### Chronic venous disease (CVD)

The descriptive measures of age and BMI do not suggest an association of these variables with CEAP. The median Child-Pugh for patients classified in classes C4 to C6 (9.00) is higher than the median for patients classified in classes C0 to C3 (7.00).

The descriptive measures of the Meld Score also do not suggest an association of this variable with CEAP, and we found that as the severity of ascites increases, there is an increase in the proportion of patients classified in the category indicating more severe clinical manifestations of chronic venous disease (C4 to C6). The percentage of patients classified in classes C4 to C6 in those without hernia is lower than in those with hernia, and the highest percentage of patients classified in classes C4 to C6 was observed in those who remained standing (orthostatism) for more than 6 h.

The joint behavior of the candidate variables for risk factors for chronic venous insufficiency and the VCSS–Venous Clinical Severity Scale is shown in Table 4. The Spearman correlation coefficient between VCSS and age is 0.123, indicating no association between the variables and the same for body mass index, with the value of Spearman’s correlation coefficient of 0.075. It is observed that as the value of VCSS increases, Child-Pugh tends to increase.

**TABLE 4:** Factors impacting VCSS score- descriptive measures.

Child-Pugh	*n*	Mean	Std derivation	Min	1° quartile	Median	3° quartile	Max
**Venous clinical severity scale by child-Pugh–Liver cirrhosis severity scale**								
A (5–6)	31	5.03	4.01	0.0	2.5	5.0	6.5	19
B (7–9)	69	7.28	4.54	0.0	4.0	6.0	10	20
C (10–15)	19	7.53	3.52	1.0	4.0	8.0	10	14
**Ascites**	***n***	**Mean**	**Std derivation**	**Min**	**1° quartile**	**Median**	**3° quartile**	**Max**
**Venous clinical severity scale for ascites**								
Absence	23	6.13	4.49	0.0	3.0	5.0	8.5	16
Low/mild	40	6.45	4.87	0.0	3.0	5.0	10	19
Bulky/refractary	56	7.18	3.90	1.0	4.0	6.5	10	20
**Ortostatism**	***n***	**Mean**	**Std derivation**	**Min**	**1° quartile**	**Median**	**3° quartile**	**Max**
**Orthostatism venous clinical severity scale**								
Less than 3 h	10	4.50	3.34	0.0	3.25	4.0	5.75	11
Between 3 and 6 h	35	5.77	3.72	0.0	3.50	5.0	8.00	14
More than 6 h	73	7.47	4.61	0.0	4.00	6.0	7.47	10

The Spearman correlation coefficient between the Meld Score scale and VCSS was 0.078, indicating no association between the two scales.

The median of the VCSS scale observed in patients with refractory ascites is higher than that observed in the other grades of ascites.

Descriptive measures of the VCSS scale observed in the groups with and without hernias do not suggest an association between these variables. The mean and median of the VCSS scale increase with increasing orthostatism, and descriptive measures of the presence of an extracardiac shunt on transthoracic echocardiography also do not suggest an association of this condition with VCSS. Table 5 shows the descriptive measures for the severity scales of liver disease, chronic venous disease VCSS. Remembering that this scale varies between 0 and 30, with increasing severity, at least 50.0% of the patients have a VCSS scale value below.

**TABLE 5:** Descriptive measures for MELD Score and VCSS.

	*N*	Mean ± SD	Median (Q1–Q3)	Min–Max
MELD	119	14.77 ± 4.50	15.00 (11.00–18.00)	6.00–25.00
VCSS	119	6.73 ± 4.34	6.00 (4.00–10.00)	0.00–20.00

The mean age (54 years) was higher in patients with DVT than in those without. The mean BMI of patients without DVT (25.7 kg/m^2^) is lower than that of patients with DVT (27.0 kg/m^2^). The prevalence of DVT is higher in patients with thrombophilia (20.0%) than in those without (7.0%). This suggests an association between the two variables (Table 6). The descriptive measures of the MELD score, the cirrhosis scale used for liver transplant waiting lists, did not indicate an association of this scale with the occurrence of DVT.

**TABLE 6:** Descriptive measures for age, BMI and MELD for DVT.

		*N*	Mean ± SD	Median (Q1–Q3)	Min–Max
Age	No DVT	110	53.99 ± 13.48	57.00 (46.00–64.00)	21.00–76.00
	DVT	9	54.11 ± 10.20	55.00 (46.00–59.00)	38.00–70.00
BMI	No DVT	106	27.06 ± 5.07	25.71 (23.08–30.40)	19.50–40.39
	DVT	9	27.07 ± 4.07	27.03 (24.00–28.65)	21.22–35.15
MELD score	No DVT	110	14.85 ± 4.51	15.00 (11.25–18.00)	6.00–25.00
	DVT	9	13.89 ± 4.51	15.00 (9.00–18.00)	8.00–20.00

The prevalence of DVT can be considered high in all etiologies of cirrhosis except those related to the hepatitis B virus.

### Chronic venous disease risk factors identification

We evaluated possible associations of chronic venous insufficiency with the following variables that may be risk factors for the disease: age, BMl, Child-Pugh, MELD score, ascites, hernia, orthostatism. The joint behavior of the candidate variables for risk factors for chronic venous insufficiency and the categorized CEAP is illustrated in Table 7. The descriptive measures of age and BMI do not suggest an association of these variables with CEAP. Orthostatism was evaluated according to the patient’s report related to his daily habits during life, especially at work, evaluating the time in which he was mainly standing or with the limbs hanging without walking. Ranged from <3 h, 3–6 h and >6 h.

**TABLE 7:** Descriptive measures for age and CEAP, BMI for CEAP, and Child-Pugh for CEAP.

		*N*	Mean ± SD	Median (Q1–Q3)	Min–Max
Age	C0–C3	81	53.47 ± 13.78	57.00 (44.00–64.00)	21.00–75.00
	C4–C5	38	55.13 ± 12.06	56.00 (46.50–63.00)	28.00–76.00
BMI	C0–C3	78	27.11 ± 5.25	25.75 (22.89–30.85)	19.50–40.39
	C4–C5	37	26.96 ± 4.75	25.70 (23.24–29.28)	20.28–39.44
Child-Pugh	C0–C3	81	7.59 ± 1.63	7.00 (6.00–9.00)	5.00–11.00
	C4–C5	38	8.32 ± 1.61	9.00 (7.00–9.00)	5.00–11.00

The mean Child Pugh for patients classified in classes C4 to C6 (9.00) is higher than the mean for patients classified in classes C0 to C3 (7.00).

Descriptive measures of the MELD score also do not suggest an association of this variable with CEAP. As the severity of ascites increases, the proportion of patients classified as having more severe clinical manifestations of chronic venous disease (C4 to C6) increases.

The percentage of patients classified in categories C4 to C6 is lower in those without hernia than in those with hernia. The highest percentage of patients classified in category C4 to C6 was observed in those who remained standing (orthostatism) for more than 6 h. The joint behavior of the candidate variables for risk factors for chronic venous insufficiency VCSS—venous clinical severity scale and MELD is illustrated in Table 8.

**TABLE 8:** Descriptive measures of MELD and VCSS.

	*N*	Mean ± SD	Median (Q1–Q3)	Min–Max
MELD	119	14.77 ± 4.50	15.00 (11.00–18.00)	6.00–25.00
VCSS	119	6.73 ± 4.34	6.00 (4.00–10.00)	0.00–20.00

Multiple linear regression models with VCSS as the response variable and risk factors as explanatory variables. In the adjustment of the model, the automatic procedures of selection of forward and backward variables were adopted. The variables Child-Pugh, Edema and Orthostatism were selected to compose the final model by both methods. The adjusted model is presented in Table 9.

**TABLE 9:** Results obtained in the adjustment process of the linear regression model with VCSS response variable and the risk factors for CVI as explanatory.

	Estimates of coefficients	Standard error	*P*-value
Intercept	3.698	2.534	0.147
Child-Pugh B	3.037	1.549	0.052
Child-Pugh C	2.743	2.141	0.203
Edema	7.411	1.332	<0.001
Orthostatism 1	3.372	2.559	0.190
Orthostatism 2	4.573	2.398	0.059

Selected variables.

Fixed edema and the degree of orthostatism for patients classified as Child-Pugh B, an increase of 3.037 on the VCSS is expected, on average, in relation to those classified as Child-Pugh A; for patients classified as Child-Pugh C, a mean increase of 2.743 on the VCSS scale is expected in relation to those with Child-Pugh A.

The associations of the VCSS with the quantitative variables, Age, BMI and Meld Score were evaluated by calculating the Spearman correlation coefficient. The values of the coefficients and corresponding *p*-values presented in Table 10 indicate that there is no correlation between Villalta and the quantitative variables individually.

**TABLE 10:** Spearman correlation coefficient of the VCSS with age, BMI and MELD.

	Statistics	*p*-value
Age	253,682	0.296
BMI	228,462	0.294
MELD	266,024	0.569

## Discussion

In this study, the collection of history, questionnaires, and physical examination were performed in the field, which represents an advantage in relation to other studies published on this topic, with a much larger number of patients, but using an administrative database, based on the International Classification of Diseases (ICD) (30). This represents an important bias since such databases do not have the primary objective of serving the research and with large heterogeneity in the population studied, making it difficult to perform meta-analyses. Qi et al. (4) in their systematic review showed that about 1% of patients with liver diseases develop or are diagnosed with VTE during their hospitalizations. However, the epidemiological data are very heterogeneous among studies. They demonstrated that the incidence of VTE appeared to be higher in studies including fewer than 1,000 patients than in those including more than 1,000 patients.

The cirrhotic patient is often malnourished, with great loss of muscle mass, decreased calf muscle pump, and decreased venous return. This may contribute to the development of venous disease in the lower limbs (31, 32). The analysis of the degree of chronic venous insufficiency associated with cirrhosis is a novelty based on hypotheses that can be partially validated by the data found. However, it’s clear that studies with a larger number of patients, controlled and using methods such as plethysmography and accurate analyses of the degree of sarcopenia, would better confirm such hypotheses. Laboratory findings of albumin and platelets in cirrhotic cases do not show the same pattern of findings in the literature, but it should be remembered that patients in this study are outpatients, and these studies are mostly inpatient. The prevalence of detected DVT and degree of CVD are consistently higher than in the general population. In 119 patients evaluated in this study, the prevalence of previous VTE was 7.6%. Dabbagh et al. (33), during 7 years of evaluation, included 190 patients, and 12 had VTE, or 6.3%, in hospitalized patients. Barba et al. (34) found 2.7% VTE in 157,654 patients. They were all hospitalized.

Bogari et al. (35) reported 18 cases of VTE, or 11%, in a total of 163 patients with severe liver disease. However, this was a sample of critically ill patients admitted to the hospital. The prevalence of VTE was 7.6, 8.1, and 8.2% in patients without cirrhosis, compensated cirrhosis, and decompensated cirrhosis, respectively, in a study by Nguyen et al. (36). In the present study, 9.7% were found to be compensated (CTP A), and 12.5% were found to be decompensated (CTP B and C). Stine considered the presence of NASH *per se* as a hypercoagulable state due to the associated inflammatory state, and in his study, the most common etiologies of cirrhosis were NASH (38%), HCV (26%), and alcoholism (24%). Among the patients admitted with liver dysfunction, 7% had a VTE, but portal vein thrombosis was also included in this article (37). The worst degrees of cirrhosis would be associated with higher CVD and DVT rates. Prophylactic measures could be taken in patients on the transplant list once these poor prognostic factors are established. The literature lacks studies evaluating the degree of chronic venous insufficiency in cirrhotic patients.

### Study limitations

The study is cross-sectional, and therefore it is not possible to evaluate the evolution of the chronic venous disease in the same patient together with the concomitant worsening of the cirrhosis. It is only possible to observe the patient at a given moment and thus to correlate the venous and hepatic picture. Chronicity, subjectivity, and periodicity characterize the complaints of chronic venous disease. The questionnaires used, such as the VCSS scale, may have measures that are confused with those of cirrhosis. For example, edema is common in cirrhosis and venous disease, paresthesia may occur due to alcoholic neuropathy in patients with cirrhosis, and pruritus may occur in advanced stages of CVD but also in liver disease with jaundice. Spasms are very common in cirrhotic patient with ascites using diuretics, and their occurrence in chronic venous disease is considered in the VCSS scale.

Patients with portal vein thrombosis often, but not always, use oral anticoagulants or subcutaneous enoxaparin, which may reduce DVT occurrence in this population.

## Conclusion

The incidence of VTE and CVI within the sample surpassed that of the general population; nevertheless, more studies are required to validate these results.

Concerning venous thromboembolism, no correlation was observed between the variables within the sample and the augmented risk of VTE. Regarding chronic venous disease, studies have shown that edema and orthostatism are correlated with increased severity of CVD on the VCSS scales. Regarding chronic venous disease, studies have shown that edema and orthostatism are correlated with increased severity of CVD on the VCSS scales. Statistical dispersion methods suggest that patients with higher BMI and more severe liver disease (according to the Child-Pugh score) are more likely to experience worsening of CVD.

About chronic venous disease, studies have shown that edema and orthostatism are correlated with increased severity of CVD on the VCSS scales.

## Data availability statement

The original contributions presented in this study are included in the article/Supplementary material. Further inquiries can be directed to the corresponding author.

## Author contributions

LC, FT, and WA contributed to conception and design of the study. LC, DW, VS, and RP organized the database. LC, RM, and LN performed the statistical analysis. LC wrote the first draft of the manuscript. LC, LD, RD, AT, and LD’A wrote sections of the manuscript. All authors contributed to manuscript revision, read, and approved the submitted version.
