# Supplementary material for: Prevalence of chronic venous insufficiency and deep vein thrombosis in cirrhotic patients
Source: Front Med (Lausanne). 2023 Sep 27;10:1214517. doi: 10.3389/fmed.2023.1214517 (PMC10565485; doi:10.3389/fmed.2023.1214517)
Supplement: Supplementary file 1 [file Table_1.pdf]

VCSS

ATTRIBUTE	ABSENT = 0	MILD = 1	MODERATE = 2	SEVERE = 3
**PAIN**	NONE	OCCASIONAL, NOT REQUIRE ANALGESICS OR LIMIT ACTIVITY	DAILY, MODERATE ACTIVITY LIMITATION, OCCASIONAL ANALGESICS	DAILY, SEVERE LIMITING ACTIVITIES OR REQUIRING REGULAR USE OF ANALGESICS
**VARICOSE VEINS**	NONE	FEW, SCATTERED: BRANCH VARICOSE VEINS	MULTIPLE: GREATER SAPHENOUS VARICOSE VEINS CONFINED TO CALF OR THIGH	EXTENSIVE: THIGH AND CALF OR GREATHER SAPHENOUS AND LESSER SAPHENOUS DISTRIBUTION.
**VENOUS EDEMA**	NONE	EVENING ANKLE EDEMA ONLY	AFTERNOON EDEMA, ABOVE ANKLE	MORNING EDEMA ABOVE ANKLE AND REQUIRING ACTIVITY CHANGE, ELEVATION
**SKIN PIGMENTATION**	NONE OR LOCAL, LOW INTENSITY	DIFFUSE, BUT LIMITED IN AREA AND OLD(BROWN)	DIFFUSE OVER MOST OF GAITER DISTRIBUTION (LOWER 1/3) OR RECENT PIGMENTATION(PURPLE)	WIDER DISTRIBUTION (ABOVE LOWER 1/3) AND RECENT PIGMENTATION
**INFLAMMATION**	NONE	MILD CELLULITIS, LIMITED TO MARGINAL AREA AROUND ULCER	MODERATE CELLULITIS, INVOLVES MOST OF GAITER AREA (LOWER 1/3)	WIDER DISTRIBUTION (ABOVE AND IN LOWER 1/3) OR SIGNIFICANT VENOUS ECZEMA
**INDURATION**	NONE	FOCAL, CIRCUMMALLEOLAR (<5 CM)	MEDIAL OR LATERAL, LESS THAN LOWER THIRD OF LEG	ENTIRE LOWER THIRD OF LEG OR MORE
**NUMBER OF ACTIVE ULCERS**	0	1	2	>2
**ACTIVE ULCERATIO DURATION**	NONE	< 3 MONTHS	>3 MONTHS, < 1 YEAR	NOT HEALED > 1 YEAR
**ACTIVE ULCER SIZE**	NONE	<2CM DIAMETER	2 TO 6 CM DIAMETER	>6 CM DIAMETER
**COMPRESSIVE THERAPY**	NOT USED OR NOT COMPLIANT	INTERMITTENT USE OF STOCKINGS	WEARS ELASTIC STOCKINGS MOST DAYS	FULL COMPLIANCE

CEAP classification
C0- without visible or palpable venous disesase
C1- reticular veins, phlebectasic corona, telangiectasis
C2- Varicose vein
C3- Edema
C4 – lipodermatoschlerosys, venous eczema, hyperpigmentation
C5- healed ulcer
C6- active ulcer
